# Serum autophagy protein 5 is positively related to T helper 2/T helper 1 ratio, inflammation, and exacerbation in adult asthma patients

**DOI:** 10.1186/s13223-023-00821-3

**Published:** 2023-08-29

**Authors:** Changjiang Ke, Sheng Xie

**Affiliations:** 1 Department of Pulmonary and Critical Care Medicine, Huangshi Central Hospital, Affiliated Hospital of Hubei Polytechnic University, No. 141 Tianjin Road, Huangshi, 435000 People’s Republic of China; 2 Hubei Key Laboratory of Kidney Disease Pathogenesis and Intervention, Huangshi, Hubei, 435000 People’s Republic of China

**Keywords:** Autophagy protein 5, Asthma, T helper cell, Inflammation, Exacerbation

## Abstract

**Background:**

Autophagy protein 5 (ATG5) regulates airway epithelial cell autophagy, immune response, and inflammation, which is involved in asthma progression. This study aimed to evaluate ATG5 levels and its clinical roles in adult asthma patients.

**Methods:**

Totally, 200 adult asthma patients and 100 healthy controls (HCs) were enrolled in this case-control study. Subsequently, serum ATG5 was measured by enzyme-linked immunosorbent assay.

**Results:**

ATG5 was increased in asthma patients compared with HCs [median (interquartile range): 44.2 (31.7–77.8) vs. 23.2 (16.7–39.2) ng/mL] (*P* < 0.001). In asthma patients, ATG5 was positively related to male gender (*P* = 0.022), a family history of asthma (*P* = 0.035), eosinophil count (*P* < 0.001), and immune globulin E (*P* < 0.001), while it was negatively correlated with forced expiratory volume in 1 s (FEV_1_)/forced vital capacity (*P* < 0.001) and FEV_1_ (Predicted) (*P* < 0.001). Meanwhile, ATG5 was inversely associated with T helper (Th) 1 cells (*P* = 0.008), while it was positively linked with Th2 cells (*P* < 0.001), Th2/Th1 ratio (*P* < 0.001), interleukin (IL)-4 (*P* = 0.002), and IL-4/interferon-γ ratio (*P* = 0.015). Additionally, ATG5 was positively correlated with tumor necrosis factor-α (*P* < 0.001), IL-1β (*P* = 0.001), IL-6 (*P* = 0.003), and IL-17 (*P* = 0.029). Notably, ATG5 was elevated in asthma patients at exacerbation compared to those at remission [median (interquartile range): 53.6 (37.6–90.0) vs. 35.6 (28.2–51.5) ng/mL] (*P* < 0.001). It was also noteworthy that ATG5 was positively linked with exacerbation severity in asthma patients (*P* = 0.005).

**Conclusion:**

Serum ATG5 is related to increased Th2/Th1 ratio, inflammation, exacerbation risk and severity in adult asthma patients, which serves as a candidate marker for the management of asthma. However, further validation is still needed.

**Supplementary Information:**

The online version contains supplementary material available at 10.1186/s13223-023-00821-3.

## Background

Asthma is characterized by airway obstruction, bronchial hyper-responsiveness, and airway inflammation, resulting in a persistent cough, dyspnea, and chest tightness [1, 2]. Generally, asthma mainly occurs in child patients, and it is prone to transit to adult asthma due to the long course and poor control of the disease [3, 4]. For asthma patients, the key to treatment includes long-term control to reduce the frequency and extent of disease attack [5]. Although some progress has been made in the treatment of asthma in recent years, some patients still have a poor quality of life and even die [6–8]. Therefore, it is critical to search for a potential marker for the management of asthma.

Several factors are reported to be linked with the pathogenesis of asthma, such as dysregulation of the immune response driven by cluster of differentiation 4^+^ (CD4^+^) T helper (Th) cells, airway inflammation, airway hyperresponsiveness, and airway remodeling [9–11]. Autophagy protein 5 (ATG5), one of the key proteins regulating autophagy activity, is considered to play an important role in the pathogenesis of asthma by regulating Th cell differentiation, inflammation, airway epithelial cell autophagy, and airway remodeling [12–14]. For example, one study shows that ATG5 skews the Th2/Th1 balance toward increased levels of Th2 cells, which causes airway inflammatory reactions and accelerates the development of asthma [12]. Another study indicates that ATG5 promotes airway smooth muscle cell (ASMC) inflammation, fibrosis, and autophagy, leading to asthma [13]. Moreover, one study suggests that suppressing ATG5 inhibits autophagy in bronchial epithelial cells, which reduces airway fibrosis and airway remodeling, thus alleviating asthma [14]. The above studies reveal the potential of ATG5 as a marker for asthma management. However, the previous studies have only shown an abnormal expression of ATG5 in asthma children [13, 15]. Moreover, the clinical roles of ATG5 in adult asthma patients have not been explored.

Therefore, this study aimed to evaluate the ATG5 level and its correlation with the Th2/Th1 ratio, inflammatory cytokines, exacerbation risk and severity in adult asthma patients.

## Methods

### Subjects

This case-control study consecutively enrolled 200 asthma patients and 100 healthy controls (HCs) between July 2021 and May 2022. Patients were included if they (i) followed the diagnostic criteria for asthma in adults according to the Global Initiative for Asthma (GINA) guideline [16]; (ii) were ≥ 18 years of age; (iii) were free of systemic autoimmune diseases, cancer, and hematologic malignancies. HCs would be included if they: (i) were ≥ 18 years of age; (ii) had no abnormalities on physical examination; (iii) had no history of allergic diseases, such as allergic rhinitis, or asthma. This research protocol was approved by the Ethics Committee of Huangshi Central Hospital [K2018015-R]. Patients completed the signature of informed consent.

### Data Collection

In this study, the clinical characteristics of all subjects were collected and analyzed, including demographics, eosinophil count, immune globulin E (IgE), forced expiratory volume in 1 s (FEV_1_)/forced vital capacity (FVC), and FEV_1_ (Predicted). Of these, age, gender, family history of asthma, and history of allergic rhinitis in demographics were collected from all subjects, whereas disease status and treatment information during the enrollment were collected from asthma patients. Eosinophil count was measured by means of eosin staining. IgE levels were assessed by enzyme-linked immunosorbent assay (ELISA) using Human IgE ELISA Kits (Cat. No. ab195216; sensitivity 0.02 ng/mL; detection range: 0.11–1.33 ng/ml). FEV_1_ and FVC were measured through a MIR Spirolab (Cosmed, Italy). The definition of asthma remission was the absence of symptoms and attacks, and the optimization of lung function [17]. The definition of asthma exacerbation was an acute or subacute attack with progressive worsening symptoms such as chest tightness, and cough [18]. The severity of exacerbation in asthma patients was assessed per the guidelines for the Diagnosis and Management of Asthma [19].

#### Sampling

Peripheral blood samples were collected from all included subjects after enrollment (M0), as well as from asthma patients at 1 month (M1) and 3 months (M3) after treatment. Then, serum samples were isolated and stored. The ATG5 levels in serum samples were measured with Human ATG5 ELISA assay kits (Clone Cloud, USA; Cat. No. SEL221Hu; sensitivity 0.29 ng/mL; detection range: 0.78-50 ng/mL) by ELISA. The levels of Th1 and Th2 cells in 125 asthma patients were measured with the Human Th1/Th2 Phenotyping Kit (BD Biosciences, USA; Cat. No. 560,751) by flow cytometry (FCM). Then, the levels of interleukin-4 (IL-4), interferon-gamma (IFN-γ), tumor necrosis factor-α (TNF-α), interleukin-1β (IL-1β), interleukin-6 (IL-6), and interleukin-17 (IL-17), were detected among 200 asthma patients with Human ELISA Kits (Abcam, UK). The kits used were as follows: Human IL-4 ELISA Kit (Cat. No. ab46058; sensitivity 0.5 pg/mL; detection range: 1.1–35 pg/ml), Human IFN-γ High Sensitivity ELISA Kit (Cat. No. ab46048; sensitivity 0.69 pg/mL; detection range: 0.78-25 pg/ml), Human TNF-α ELISA Kit (Cat. No. ab181421; sensitivity 4.32 pg/mL; detection range: 15.63–1000 pg/ml), Human IL-1β High Sensitivity ELISA Kit (Cat. No. BMS224HS; sensitivity 0.05 pg/mL; detection range: 0.16-10.0 pg/ml), Human IL-6 ELISA Kit (Cat. No. ab178013; sensitivity 1.6 pg/mL; detection range: 7.8–500 pg/ml), Human IL-17 ELISA Kit (Cat. No. ab119535; sensitivity 0.5 pg/mL; detection range: 1.6–100 pg/ml).

### Detection

For FCM, the experimentation was strictly according to the kit instruction. In brief, T helper cells were stained using specific fluorescent antibodies (CD4, IFN-γ and IL-4) after stimulation, then were counted using a fluorescence activated cell sorting (FACS) flow cytometer. Following that, the proportions of Th1 and Th2 cells in CD4^+^ T cells were imputed.

The procedures of ELISA were in strict accordance with the instructions. Briefly, 50 µL of standards, control, or samples were added to each well, which was incubated for 2 h. Following that, 50 µL of conjugated antibody was added and incubated for 1 h. Subsequently, 100 µL of substrate solution was added and incubated for 30 min. Finally, 100 µL of stop solution was added, then absorbance was read at 450 nm immediately. Standard curve was fitted and used to calculate the concentration of samples.

### Statistics

Normality determination for continuous variables was performed using the Kolmogorov-Smirnov test. For the normal distributed continuous data, they were displayed as mean ± standard deviation (SD); for the skewed distributed continuous data, they were presented as median and inter-quartile range (IQR); for the categorized data, they were expressed as count (percentage). A comparison of the clinical characteristics of the two groups was conducted using the Wilcoxon rank sum test (for skewed distributed continuous data), χ^2^ test (for categorized data), or Student’s t-test (for normally distributed continuous data). The Wilcoxon rank sum test was employed to measure the disparity in ATG5 levels between the two groups. The receiver-operating characteristic (ROC) curve was employed to demonstrate the capacity of ATG5 to discriminate asthma patients from HCs and patients in exacerbation from those in remission. Spearman’s rank correlation test was employed to evaluate the relationship between the two variables. The results were statistically significant when *P* < 0.05.

## Results

### Baseline characteristics of adult asthma patients and HCs

The adult asthma patients included 89 (44.5%) females and 111 (55.5%) males with a mean age of 28.9 ± 8.0 years. Meanwhile, the HCs included 40 (40.0%) females and 60 (60.0%) males with a mean age of 30.1 ± 8.2 years. There was no difference in age (*P* = 0.227) or gender (*P* = 0.458) between adult asthma patients and HCs. In addition, the rates of a family history of asthma (*P* = 0.014) and a history of allergic rhinitis (*P* < 0.001) were higher in adult asthma patients than in HCs. Besides, the median values of eosinophil count [median (IQR): 0.3 (0.2–0.6) vs. 0.1 (0.1–0.1) 10^9^/L] and IgE [median (IQR): 151.5 (79.0-287.0) vs. 36.5 (23.3–51.8) IU/mL] were higher in adult asthma patients than those in HCs (both *P* < 0.001). Furthermore, the mean levels of FEV_1_/FVC (mean ± SD: 71.8 ± 7.8% vs. 84.1 ± 3.6%) and FEV_1_ (Predicted) (mean ± SD: 81.9 ± 8.1% vs. 99.5 ± 6.3%) were lower in adult asthma patients than those in HCs (both *P* < 0.001). Additionally, in adult asthma patients, the median (IQR) values of Th1 cells, Th2 cells, and the Th2/Th1 ratio were 14.0 (11.4–17.2)%, 15.3 (12.6–19.1)%, and 1.2 (0.8–1.5), respectively. More specific clinical characteristics of all participants were described in Table 1.

**Table 1:** Clinical characteristics

Items	HCs	Asthma patients	*P* value
	(N = 100)	(N = 200)	
Age (years), mean ± SD	30.1 ± 8.2	28.9 ± 8.0	0.227
Gender, No. (%)			0.458
Female	40 (40.0)	89 (44.5)	
Male	60 (60.0)	111 (55.5)	
Family history of asthma, No. (%)			0.014
No	88 (88.0)	152 (76.0)	
Yes	12 (12.0)	48 (24.0)	
History of allergic rhinitis, No. (%)			< 0.001
No	100 (100.0)	59 (29.5)	
Yes	0 (0.0)	141 (70.5)	
Eosinophil count (10^9^/L), median (IQR)	0.1 (0.1–0.1)	0.3 (0.2–0.6)	< 0.001
IgE (IU/mL), median (IQR)	36.5 (23.3–51.8)	151.5 (79.0-287.0)	< 0.001
FEV_1_/FVC (%), mean ± SD	84.1 ± 3.6	71.8 ± 7.8	< 0.001
FEV1 (predicted) (%), mean ± SD	99.5 ± 6.3	81.9 ± 8.1	< 0.001
Disease status, No. (%)			−
Remission	−	78 (39.0)	
Exacerbation	−	122 (61.0)	
Exacerbation severity, No. (%)			−
Mild	−	47 (38.5)	
Moderate	−	49 (40.2)	
Severe	−	26 (21.3)	
Treatment, No. (%)			−
Beta 2 agonists	−	148 (74.0)	
Corticosteroids	−	120 (60.0)	
Anti-histamines	−	71 (35.5)	
Allergen-specific immunotherapy	−	20 (10.0)	
Others	−	134 (67.0)	
Th1 cells (%), median (IQR)	−	14.0 (11.4–17.2)	−
Th2 cells (%), median (IQR)	−	15.3 (12.6–19.1)	−
Th2/Th1 ratio, median (IQR)	−	1.2 (0.8–1.5)	−
IFN-γ (pg/mL), median (IQR)	−	7.8 (6.1–10.3)	−
IL-4 (pg/mL), median (IQR)	−	15.0 (12.0-18.7)	−
IL-4/IFN-γ ratio, median (IQR)	−	1.9 (1.2–2.8)	−
TNF-α (pg/mL), median (IQR)	−	69.5 (53.1–92.3)	−
IL-1β (pg/mL), median (IQR)	−	2.0 (1.5–2.7)	−
IL-6 (pg/mL), median (IQR)	−	35.8 (26.7–45.6)	−
IL-17 (pg/ml), median (IQR)	−	67.6 (53.4–96.5)	−

HCs, healthy controls; SD, standard deviation; IQR, interquartile range; IgE, immunoglobulin E; FEV_1_, forced expiratory volume in 1 s; FVC, forced vital capacity; Th, T-helper; IFN-γ, interferon-gamma; IL, interleukin; TNF-α, tumor necrosis factor-alpha

### Comparison of ATG5 between adult asthma patients and HCs

**Fig. 1:**
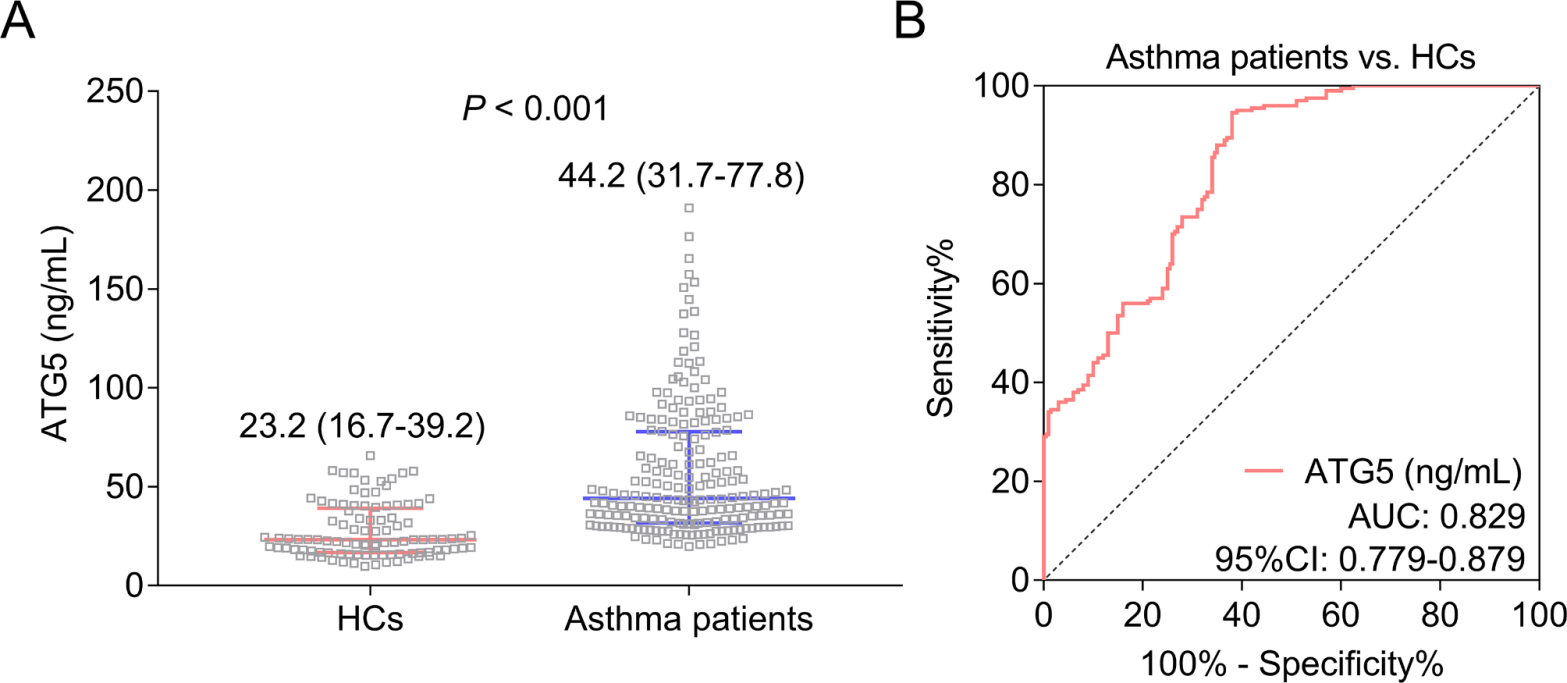
ATG5 in adult asthma patients and HCs. Comparison of ATG5 (skewed distributed continuous data) between adult asthma patients and HCs (Wilcoxon rank sum test) (**A**); ROC curve showing the ability of ATG5 (skewed distributed continuous data) to distinguish adult asthma patients from HCs (ROC curve) (**B**)

ATG5 was elevated in adult asthma patients compared with HCs [median (IQR): 44.2 (31.7–77.8) vs. 23.2 (16.7–39.2) ng/mL] (*P* < 0.001) (Fig. 1A). In addition, ATG5 disclosed a good value to distinguish adult asthma patients from HCs with area under curve (AUC) of 0.829 (95% confidence interval (CI): 0.779–0.879) (Fig. 1B).

### Correlation of ATG5 with clinical features in adult asthma patients

**Fig. 2:**
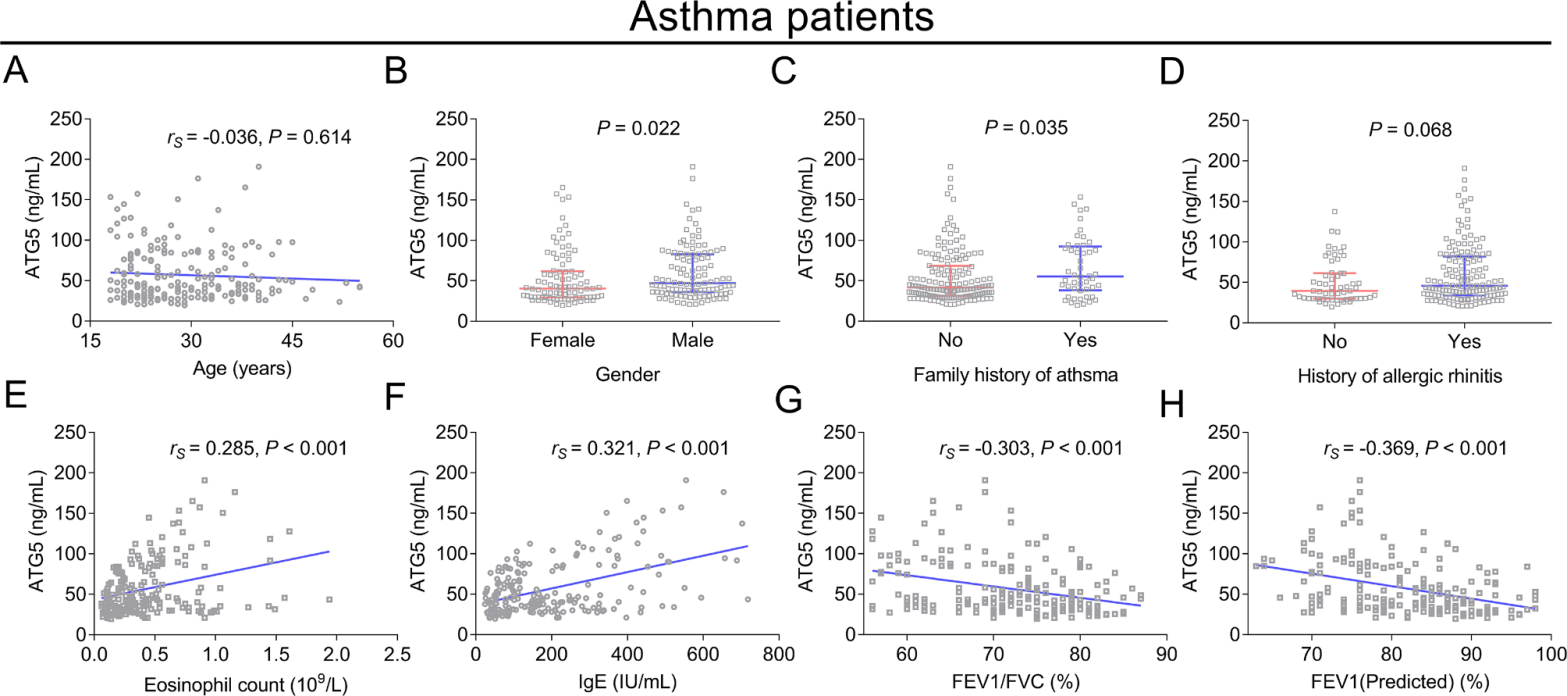
Relationship of ATG5 with clinical features in adult asthma patients. The association of ATG5 (skewed distributed continuous data) with age (normal distributed continuous data, Spearman’s rank correlation test) (**A**), gender (categorized data, Wilcoxon rank sum test) (**B**), family history of asthma (categorized data, Wilcoxon rank sum test) (**C**), history of allergic rhinitis (categorized data, Wilcoxon rank sum test) (**D**), eosinophil count (skewed distributed continuous data, Spearman’s rank correlation test) (**E**), IgE (skewed distributed continuous data, Spearman’s rank correlation test) (**F**), FEV_1_/FVC (normal distributed continuous data, Spearman’s rank correlation test) (**G**), and FEV_1_ (Predicted) (normal distributed continuous data, Spearman’s rank correlation test) (**H**) in adult asthma patients

In adult asthma patients, there was no association of ATG5 with age (*r*_*s*_=-0.036, *P* = 0.614) (Fig. 2A). However, increased ATG5 was correlated with male (*P* = 0.022) (Fig. 2B) and a family history of asthma (*P* = 0.035) (Fig. 2C). No relationship was observed between ATG5 and a history of allergic rhinitis (*P* = 0.068) (Fig. 2D). Furthermore, ATG5 was positively associated with eosinophil count (*r*_*s*_=0.285, *P* < 0.001) (Fig. 2E) and IgE (*r*_*s*_=0.321, *P* < 0.001) (Fig. 2F), while it was negatively related to FEV_1_/FVC (*r*_*s*_=-0.303, *P* < 0.001) (Fig. 2G) and FEV_1_ (Predicted) (*r*_*s*_=-0.369, *P* < 0.001) (Fig. 2H). Furthermore, ATG5 was reduced in patients with beta 2 agonists (*P* = 0.048) or allergen-specific immunotherapy (*P* = 0.016) compared to those without during the enrollment. Meanwhile, ATG5 was not different in patients with or without corticosteroids, anti-histamines, and other treatments during the enrollment (all *P* > 0.05) (Supplementary Table 1).

### Correlation of ATG5 with Th1 cells, Th2 cells, and inflammatory cytokines in adult asthma patients

**Fig. 3:**
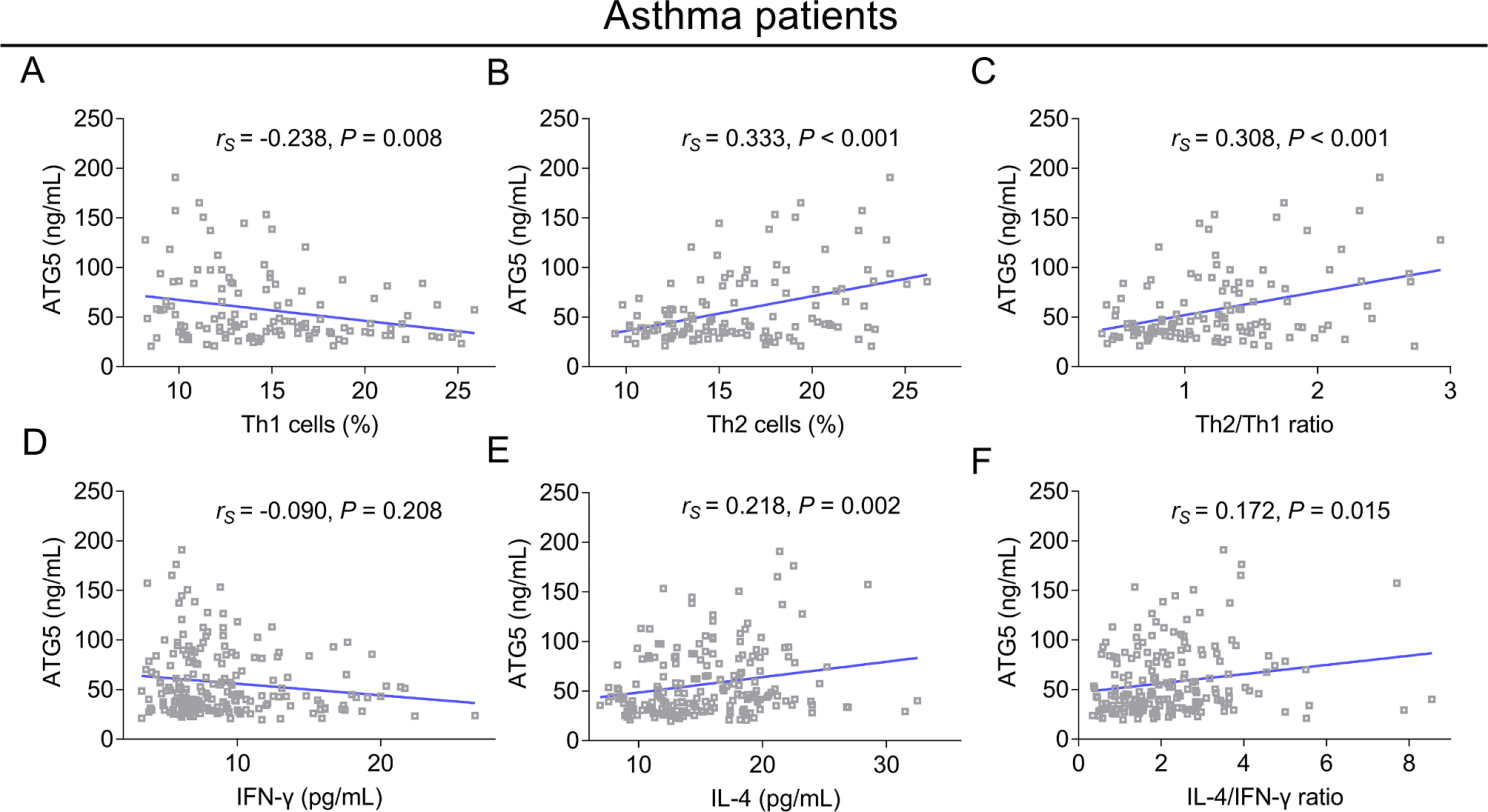
Relationship of ATG5 with Th1, Th2, and their corresponding cytokines in adult asthma patients. The association of ATG5 (skewed distributed continuous data) with Th1 cells (skewed distributed continuous data, Spearman’s rank correlation test) (**A**), Th2 cells (skewed distributed continuous data, Spearman’s rank correlation test) (**B**), Th2/Th1 ratio (skewed distributed continuous data, Spearman’s rank correlation test) (**C**), IFN-γ (skewed distributed continuous data, Spearman’s rank correlation test) (**D**), IL-4 (skewed distributed continuous data, Spearman’s rank correlation test) (**E**), and IL-4/IFN-γ ratio (skewed distributed continuous data, Spearman’s rank correlation test) (**F**) in adult asthma patients

ATG5 was inversely linked with Th1 cells (*r*_*s*_=-0.238, *P* = 0.008) (Fig. 3A), while it was positively related to Th2 cells (*r*_*s*_=0.333, *P* < 0.001) (Fig. 3B) and the Th2/Th1 ratio (*r*_*s*_=0.308, *P* < 0.001) (Fig. 3C). In addition, ATG5 was not associated with IFN-γ (*r*_*s*_=-0.090, *P* = 0.208) (Fig. 3D), but it was positively correlated with IL-4 (*r*_*s*_=0.218, *P* = 0.002) (Fig. 3E) and the IL-4/IFN-γ ratio (*r*_*s*_=0.172, *P* = 0.015) (Fig. 3F) in adult asthma patients.

**Fig. 4:**
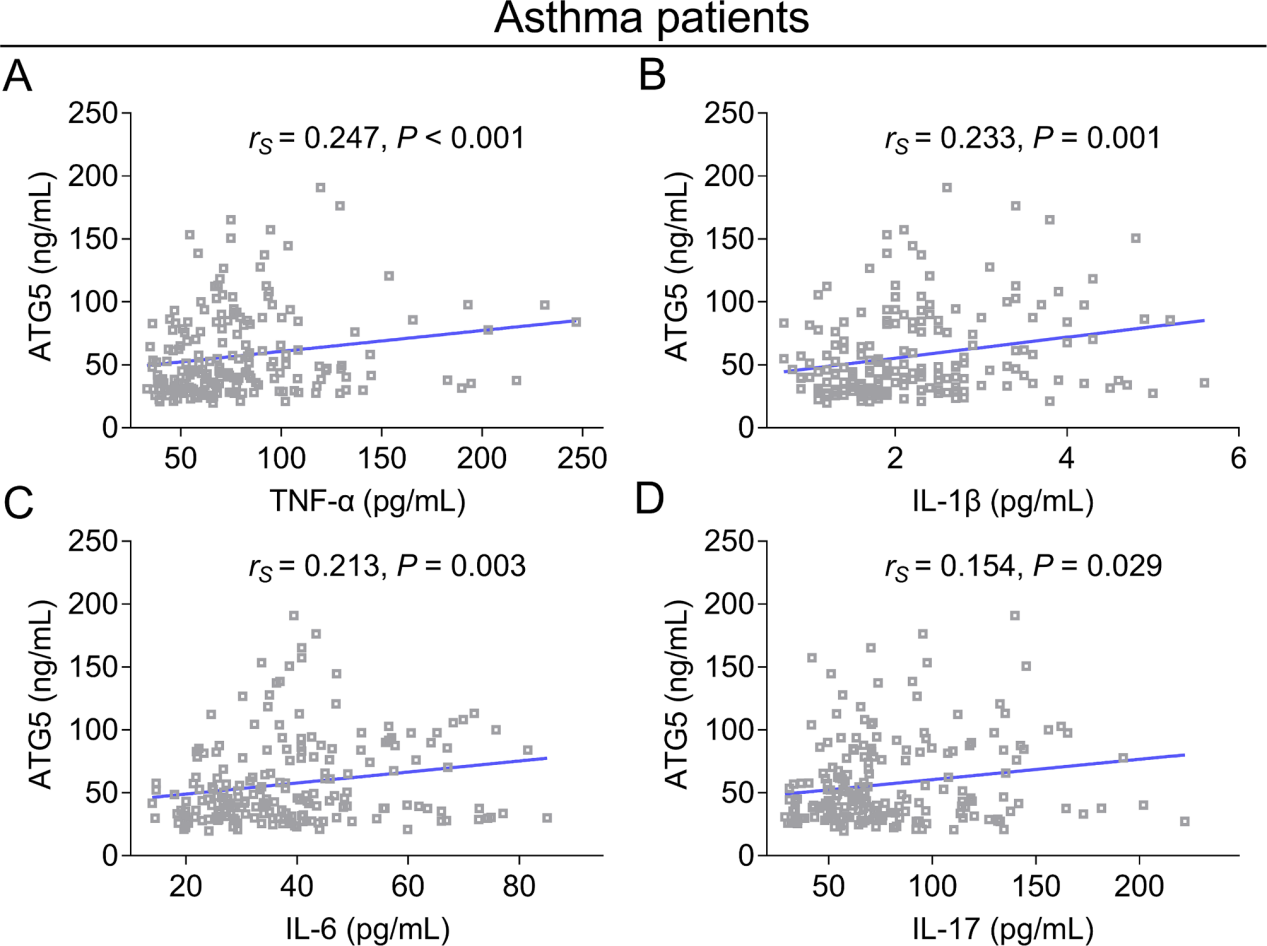
Relationship of ATG5 with inflammatory cytokines in adult asthma patients. The association of ATG5 (skewed distributed continuous data) with TNF-α (skewed distributed continuous data, Spearman’s rank correlation test) (**A**), IL-1β (skewed distributed continuous data, Spearman’s rank correlation test) (**B**), IL-6 (skewed distributed continuous data, Spearman’s rank correlation test) (**C**), and IL-17 (skewed distributed continuous data, Spearman’s rank correlation test) (**D**) in adult asthma patients

In terms of inflammatory cytokines, ATG5 was positively associated with TNF-α (*r*_*s*_=0.247, *P* < 0.001) (Fig. 4A), IL-1β (*r*_*s*_=0.233, *P* = 0.001) (Fig. 4B), IL-6 (*r*_*s*_=0.213, *P* = 0.003) (Fig. 4C), and IL-17 (*r*_*s*_=0.154, *P* = 0.029) (Fig. 4D) in adult asthma patients.

### Correlation of ATG5 with asthma exacerbation in adult asthma patients

**Fig. 5:**
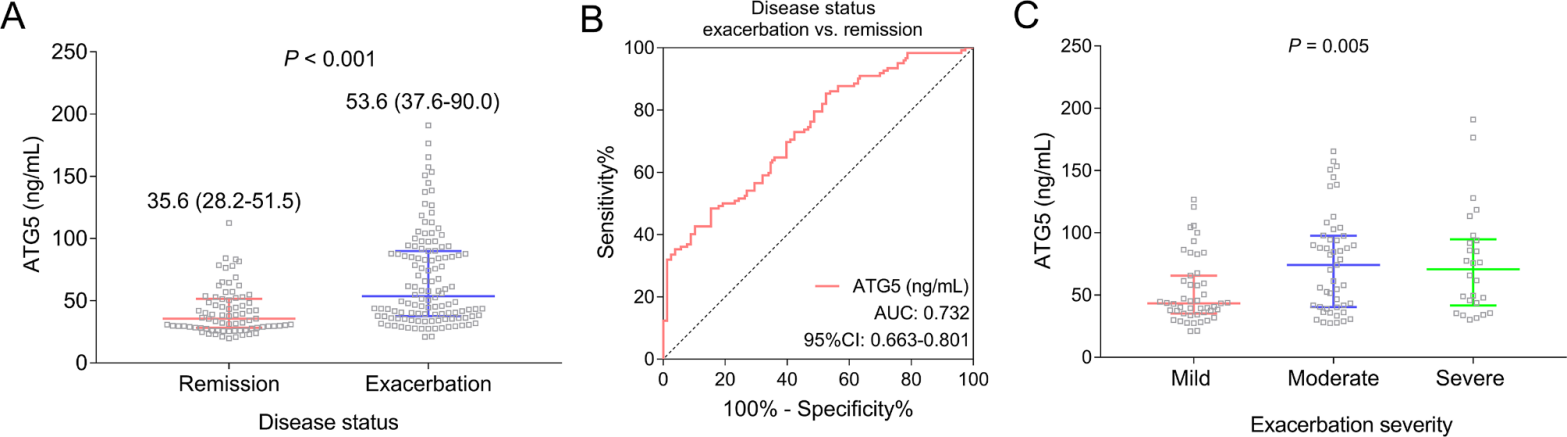
Relationship of ATG5 with exacerbation risk and severity in adult asthma patients. The association of ATG5 (skewed distributed continuous data) with exacerbation risk in adult asthma patients (categorized data, Wilcoxon rank sum test) (**A**); ROC curve showing the ability of ATG5 (skewed distributed continuous data) to discriminate adult asthma patients at exacerbation from adult asthma patients at remission (ROC curve) (**B**); the association of ATG5 (skewed distributed continuous data) with exacerbation severity in adult asthma patients (categorized data, Spearman’s rank correlation test) (**C**)

ATG5 was increased in adult asthma patients at exacerbation compared to adult asthma patients at remission [median (IQR): 53.6 (37.6–90.0) vs. 35.6 (28.2–51.5) ng/mL] (*P* < 0.001) (Fig. 5A). Meanwhile, ATG5 showed a certain value to discriminate adult asthma patients at exacerbation from adult asthma patients at remission with AUC of 0.732 (95% CI: 0.663–0.801) (Fig. 5B). Notably, ATG5 was positively linked with exacerbation severity in adult asthma patients (*P* = 0.005) (Fig. 5C). Interestingly, ATG5 was gradually reduced from M0 to M3 in adult asthma patients after treatment (*P* < 0.001) (Supplementary Fig. 1).

## Discussion

ATG5, as one of the essential proteins regulating autophagy, is considered to overexpress and mediate immune responses in asthma [12, 15, 20]. Previous reports suggest that compared with HCs, ATG5 is highly expressed in asthma children [13, 15]. However, no study has explored ATG5 level and its relationship with clinical features in adult asthma patients. Our study found that: (1) ATG5 was higher in adult asthma patients than in HCs, and it had a good value in distinguishing adult asthma patients from HCs. (2) ATG5 was positively associated with male, a family history of asthma, eosinophil count, and IgE, while it was negatively linked with FEV_1_/FVC and FEV_1_ (Predicted) in adult asthma patients.

Allergen-activated Th2 cells release cytokines, promoting airway eosinophil inflammation, airway hyperresponsiveness, and airway remodeling, which play a central role in the development of asthma [9, 21]. Notably, ATG5 participates in the pathogenesis of asthma by mediating the differentiation of CD4^+^ T cells [12, 22], which suggests that it may have a potential relationship with Th1 and Th2 cells in asthma patients. Our study revealed that in adult asthma patients, ATG5 was positively related to the Th2/Th1 ratio. The possible explanation was as follows: ATG5 could upregulate Th2 cell differentiation and inhibit Th1 cell differentiation by regulating cytokine secretion and antigen presentation, thus destroying Th2/Th1 balance in adult asthma patients [23–25]. Significantly, the detection of Th1 and Th2 cells was required to be completed on the same day after the patients’ peripheral blood sample collection. Due to the busy status of the clinical work, we were unable to detect Th1 and Th2 cells in all asthma patients. Thus, only 125 asthma patients’ Th1 and Th2 cells were detected in our study.

It is reported that ATG5 also participates in airway inflammation in asthma patients to a certain extent [26]. Therefore, the relationship between ATG5 and inflammatory cytokines in adult asthma patients is worth focusing on. In the current study, ATG5 was positively correlated with TNF-α, IL-1β, IL-6, and IL-17 in adult asthma patients. The possible reasons were as follows: (1) ATG5-mediated lung epithelial cell autophagy aggravated eosinophilic inflammation in asthma mice [27]. (2) ATG5 attenuated the inhibitory effect of miR-335-5p on the ASMC inflammatory response [13]. (3) ATG5 might participate in the inflammatory reaction of asthma through the Ras homolog gene family, member A (RhoA)/Rho-associated coiled-coil containing protein kinase (ROCK) signaling pathway [28, 29]. The above reasons indicated that ATG5 aggravated the airway inflammatory reaction in asthma patients in various ways, so it was positively correlated with inflammatory cytokines in adult asthma patients. Notably, although statistically significant, the correlations of serum ATG5 with clinical indexes (clinical features, Th1 cells, Th2 cells, and inflammatory cytokines) were relatively not strong. The reason for these results might be that we only measured ATG5 levels in serum samples, and the serum ATG5 levels might be influenced by some confounding factors (such as the patients’ own factors or disease complications), which caused some interference with the results.

Asthma has a long course, and a potential marker is needed to observe the asthma exacerbation risk and severity to control the progression of asthma [30]. Our study found that ATG5 was positively related to the exacerbation risk and severity in adult asthma patients. This might be because: (1) ATG5 mediated ASMC autophagy, which aggravated airway injury and the progression of asthma [13]. (2) ATG5 mediated the immune responses by regulating cytokine secretion and antigen presentation, and accelerated the progression of asthma [23–25](3) ATG5 regulated the inflammatory responses through the abovementioned multiple approaches and promoted the progression of asthma [13, 27]. (4) ATG5 was positively correlated with the expression of various collagen genes, leading to collagen deposition and subepithelial fibrosis, and thus aggravated the progression of asthma [31]. In summary, the ATG5 might promote the progression of asthma in the series of ways mentioned above. Therefore, ATG5 was positively associated with the exacerbation risk and severity in adult asthma patients.

There were some limitations in our study: (1) The specific mechanisms of ATG5 regulating Th1 cells, Th2 cells, and inflammatory cytokines in adult asthma patients were required to explore in future studies. (2) Our study did not include an age and sex-matched disease control group. Future research should enroll disease controls to further comprehensively verify the potential of ATG5 as a marker in adult asthma patients. (3) Although the sample size in our study was relatively large, in order to have a clearer conclusion on the clinical significance of ATG5 in adult asthma patients, a larger sample size was needed in further studies. (4) Future studies should consider collecting more direct ATG5 samples (such as respiratory epithelial samples) to assess its correlation with clinical indexes in asthma patients. (5) Our study did not evaluate the changes in ATG5 in asthma patients before the exacerbation, and further studies should consider assessing the changes in ATG5 to verify its potential as a candidate marker in asthma patients.

## Conclusions

In conclusion, serum ATG5 is positively correlated with the Th2/Th1 ratio, proinflammatory cytokines, and exacerbation in adult asthma patients, which may have important clinical significance for the management of asthma.

### Electronic supplementary material

Below is the link to the electronic supplementary material.

**Supplementary Fig. 1**: Changes of ATG5 from M0 to M3 in adult asthma patients after treatment. ATG5 (skewed distributed continuous data) was decreased continually from M0 to M3 in adult asthma patients after treatment (Friedman test)

**Supplementary Table 1**: Comparison of ATG5 in patients with different treatments during the enrollment

## Data Availability

The datasets used and/or analysed during the current study are available from the corresponding author on reasonable request.
