# Supplementary figures and images for: Serum autophagy protein 5 is positively related to T helper 2/T helper 1 ratio, inflammation, and exacerbation in adult asthma patients
Source: Allergy Asthma Clin Immunol. 2023 Aug 29;19:77. doi: 10.1186/s13223-023-00821-3 (PMC10466706; doi:10.1186/s13223-023-00821-3)

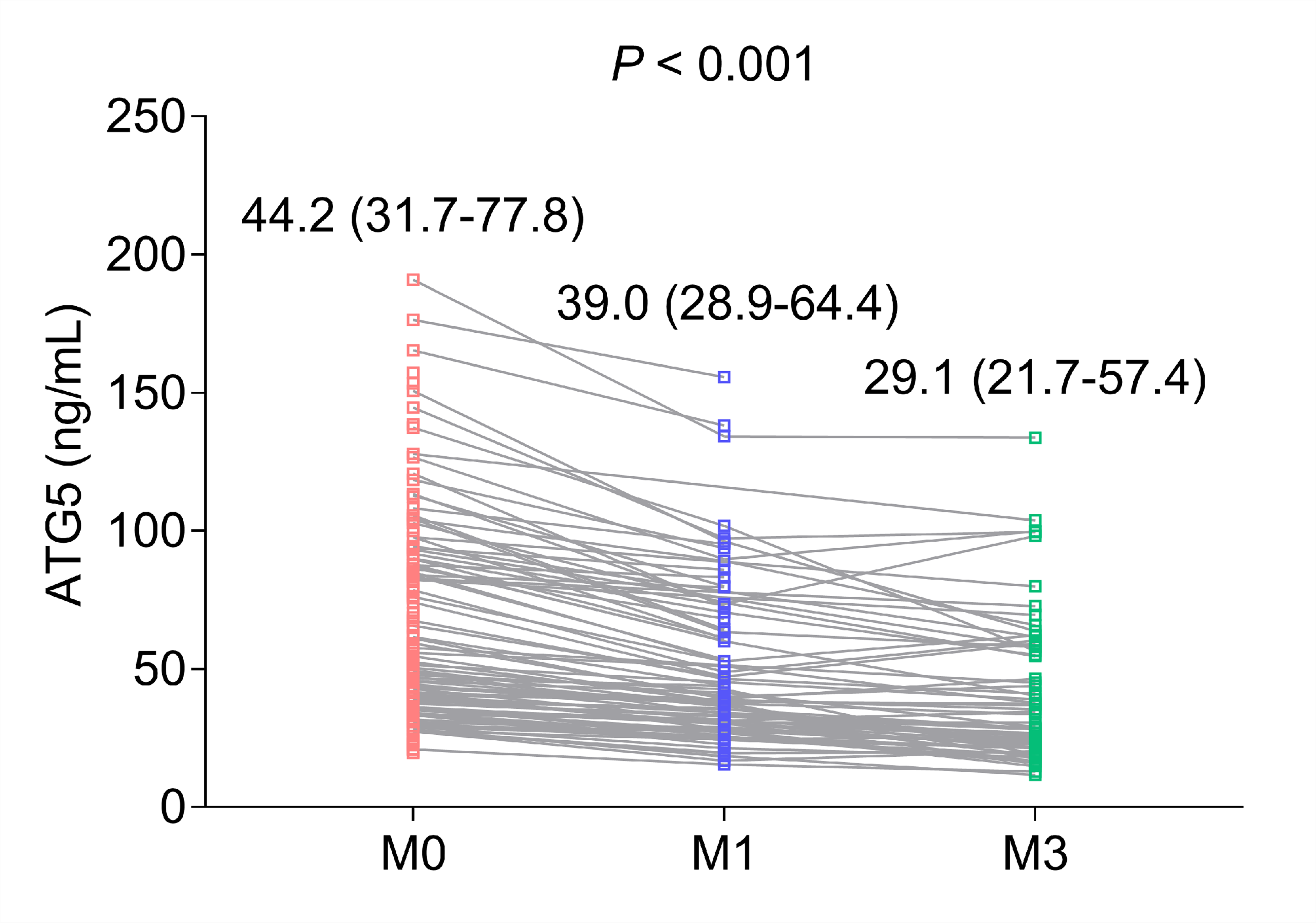

Supplement: Supplementary file 1 — Supplementary Fig. 1: Changes of ATG5 from M0 to M3 in adult asthma patients after treatment. ATG5 (skewed distributed continuous data) was decreased continually from M0 to M3 in adult asthma patients after treatment (Friedman test) [file 13223_2023_821_MOESM1_ESM.tif]
