# Supplementary material for: Serum autophagy protein 5 is positively related to T helper 2/T helper 1 ratio, inflammation, and exacerbation in adult asthma patients
Source: Allergy Asthma Clin Immunol. 2023 Aug 29;19:77. doi: 10.1186/s13223-023-00821-3 (PMC10466706; doi:10.1186/s13223-023-00821-3)
Supplement: Supplementary file 2 — Supplementary Table 1: Comparison of ATG5 in patients with different treatments during the enrollment [file 13223_2023_821_MOESM2_ESM.docx]

**Supplementary Table 1**. Comparison of ATG5 in patients with different treatments during the enrollment.

| Items | ATG5 (ng/mL), median (IQR) | *P* value |
| --- | --- | --- |
| Beta 2 agonists |  | 0.048 |
| No | 47.1 (38.0-89.4) |  |
| Yes | 43.1 (30.2-69.9) |  |
| Corticosteroids |  | 0.557 |
| No | 46.0 (32.5-78.1) |  |
| Yes | 43.1 (31.1-77.8) |  |
| Anti-histamines |  | 0.264 |
| No | 45.9 (33.6-78.6) |  |
| Yes | 40.2 (30.1-74.2) |  |
| Allergen-specific immunotherapy |  | 0.016 |
| No | 45.7 (33.5-80.9) |  |
| Yes | 33.3 (28.6-49.1) |  |
| Others |  | 0.640 |
| No | 43.4 (30.3-77.9) |  |
| Yes | 44.8 (33.1-78.1) |  |

ATG5, Autophagy protein 5; IQR, interquartile range.
